# Clinical trials using mesenchymal stem cells in liver diseases and inflammatory bowel diseases

**DOI:** 10.1186/s41232-017-0045-6

**Published:** 2017-07-03

**Authors:** Atsunori Tsuchiya, Yuichi Kojima, Shunzo Ikarashi, Satoshi Seino, Yusuke Watanabe, Yuzo Kawata, Shuji Terai

**Affiliations:** Division of Gastroenterology and Hepatology, Graduate School of Medical and Dental Science, Niigata University, 1-757 Asahimachi-dori, Chuo-ku, Niigata, 951-8510 Japan

**Keywords:** Mesenchymal stem cell, Liver disease, Inflammatory bowel disease, Cell therapy

## Abstract

Mesenchymal stem cell (MSC) therapies have been used in clinical trials in various fields. These cells are easily expanded, show low immunogenicity, can be acquired from medical waste, and have multiple functions, suggesting their potential applications in a variety of diseases, including liver disease and inflammatory bowel disease. MSCs help prepare the microenvironment, in response to inflammatory cytokines, by producing immunoregulatory factors that modulate the progression of inflammation by affecting dendritic cells, B cells, T cells, and macrophages. MSCs also produce a large amount of cytokines, chemokines, and growth factors, including exosomes that stimulate angiogenesis, prevent apoptosis, block oxidation reactions, promote remodeling of the extracellular matrix, and induce differentiation of tissue stem cells. According to ClinicalTrials.gov, more than 680 clinical trials using MSCs are registered for cell therapy of many fields including liver diseases (more than 40 trials) and inflammatory bowel diseases (more than 20 trials). In this report, we introduce background and clinical studies of MSCs in liver disease and inflammatory bowel diseases.

## Background

The digestive system, which consists of the gastrointestinal tract, liver, pancreas, and biliary tree, functions in digestion, absorption, and metabolism and affects the basis of life. Various diseases, including cancer, inflammatory disease, infection, stones, and ulcers, are studied under the context of gastroenterology. While innovative drugs against *Helicobacter pylori* [1], hepatitis C virus [2], and inflammatory bowel disease (IBD) [3] have recently been developed, there are still unmet needs in this field, including in acute and chronic liver failure and refractory IBDs. Cell therapy may fulfill these unmet needs, and cell therapies using mesenchymal stem cells (MSCs) have become a major focus in many fields [4]. MSCs are reported to have multiple functions, especially anti-fibrosis and anti-inflammatory effects are focused in acute and chronic liver failure and refractory IBDs. Furthermore, MSCs have low immunogenicity, can expand easily, and can be obtained from medical waste, suggesting their potential to expand regenerative medicine for the treatment of liver diseases and IBDs.

In this paper, we review the current status of clinical trials using autologous/allogeneic MSCs in liver diseases and IBDs.

## Characteristics of MSCs

MSCs have recently received attention as potential cell sources for cell therapy due to their ease of expansion and wide range of functions. MSCs can be obtained from not only bone marrow but also medical wastes, such as adipose tissue, umbilical tissue, and dental pulp. MSCs are positive for the common markers CD73, CD90, and CD105; however, they are negative for the endothelial marker CD31 and hematopoietic marker CD45 [4–7]. The expansion of MSCs in culture is relatively easy, and under appropriate conditions, MSCs have trilineage differentiation (osteogenic, chondrogenic, and adipogenic) potential. The effects of MSCs are broadly divided into two mechanisms: (1) recruited MSCs differentiate into functional cells to replace damaged cells, permitting the treatment of bone and cartilage damage; and (2) in response to inflammatory cytokines, MSCs help prepare the microenvironment by producing immunoregulatory factors that modulate the progression of inflammation by affecting dendritic cells, B cells, T cells, and macrophages. MSCs also produce a large amount of cytokines, chemokines, and growth factors, including exosomes, which stimulate angiogenesis, prevent apoptosis, block oxidation reactions, promote remodeling of the extracellular matrix (ECM), and induce the differentiation of tissue stem cells [4, 7, 8]. These latter mechanisms can be applied for many diseases, including liver disease and IBSs. Some studies have reported that the effects of MSCs are determined by host conditions, such as inflammation stage and the use of immunosuppressants.

Although the behaviors of MSCs after administration have been analyzed, and some studies have shown that MSCs migrate to the injured site, MSC behaviors in humans have not been fully elucidated. Some studies have reported that MSCs disappear within a few weeks and do not remain long in the target tissue [5]. Recent studies have reported that only culture-conditioned medium or exosomes induce treatment effects, suggesting that the trophic effect is the most important effect of MSCs [9–11]. Another important characteristic of MSCs is that they generally have low immunogenicity. MSCs have no antigen-presenting properties and do not express major histocompatibility complex class II or costimulatory molecules; thus, injection of autologous or allogeneic MSCs has been employed in clinical studies. Allogeneic MSC therapy has the potential to expand MSC therapy to many patients [4, 7].

## Clinical trials using MSCs

**Fig. 1:**
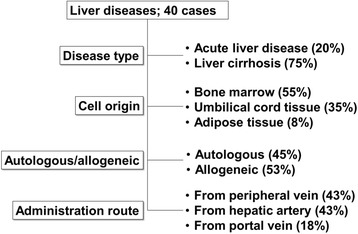
Summary of clinical trials in liver diseases

Since MSCs can be obtained relatively easily and have multiple functions, more than 680 clinical trials are ongoing according to ClinicalTrials.gov (https://clinicaltrials.gov/); most of these studies are phase I or II trials evaluating the use of MSCs in bone/cartilage, heart, neuron, immune/autoimmune, diabetes/kidney, lung, liver, and gastrointestinal fields. These studies aim to elucidate the safety/effectiveness of MSCs in the treatment of various diseases. In liver diseases, 40 trials are registered, most of which target liver cirrhosis or acute liver diseases (Table 1) [12–21]. The MSCs used in clinical trials of the liver are derived from the bone marrow (55%), umbilical cord tissue (35%), and adipose tissue (8%). Approximately 50% of MSCs are allogeneic. Additionally, while the major administration route is the peripheral blood, approximately 40% of cases are treated via the hepatic artery, reflecting the fact that hepatologists and radiologists often use catheters to treat hepatocellular carcinoma through the hepatic artery [22, 23] (Fig. 1).

**Table 1:** Clinical trials in liver diseases

No.	Start year	Cell source	Autologous/allogeneic	Administration route	Number of cells infused	Etiology	Number of patients	Follow-up period	Phase	Study design	ClinicalTrials.gov identifier	Status	Result	References
1	2013	Bone marrow	Autologous	Peripheral vein	Unknown	LC	20	48 weeks	Phase 1–2	Non-randomized, single group assignment, open label	NCT01877759	Unknown		
2	2009	Bone marrow	Autologous	Hepatic artery	5 × 106 cells/patient, 2 times	LC (alcohol)	11	24 weeks	Phase 2	Non-randomized, single group assignment, open label	NCT01741090	Unknown	Histological improvement. Improvement in Child- Pugh score. Decrease in TGFβ1, collagen type I, and α-SMA	
3	2009	Bone marrow	Autologous	Peripheral vein	1.0 × 106/kg	LC	25	24 weeks	Unknown	Non-randomized, single group assignment, open label	NCT01499459	Unknown	Improvement in Alb and MELD scores.	13
4	2014	Umbilical cord	Allogeneic	Peripheral vein	4.0 × 107/patient, 4 times	LC	320	144 weeks	Phase 1–2	Non-randomized, parallel assignment, open label	NCT01573923	Unknown		
5	2016	Adipose tissue	Autologous	Portal vein or hepatic artery	1.0 × 106/kg via peripheral vein, 3 times or 3.0 × 106/kg via hepatic artery, 3 times	LC (HCV)	5	48 weeks	Phase 1–2	Non-randomized, single group assignment, open label	NCT02705742	Recruiting		
6	2007	Bone marrow	Autologous	Peripheral or portal vein	30–50 × 106/patient	LC	8	24 weeks	Phase 1–2	Randomized, single group assignment, single blind	NCT00420134	Completed	Improvement in liver function and MELD scores.	14
7	2016	Bone marrow	Allogeneic	Peripheral vein	2.0 × 106/kg, 4 times	ACLF	30	96 weeks	Phase 1	Randomized, parallel assignment, double blind (subject, caregiver, investigator)	NCT02857010	Recruiting		
8	2009	Umbilical cord	Allogeneic	Peripheral vein	5.0 × 105/kg, 3 times	ACLF (HBV)	43	96 weeks	Phase 1–2	Randomized, parallel assignment, double blind (subject, caregiver)	NCT01218464	Unknown	Improvement in liver function and MELD scores.	15
9	2011	Bone marrow	Allogeneic	Peripheral vein	2.0 × 105/kg, 4 times or 1.0 × 106/kg, 4 times or 5.0 × 106/kg, 4 times	Liver failure (HBV)	120	48 weeks	Phase 2	Randomized, parallel assignment, open label	NCT01322906	Unknown		
10	2010	Umbilical cord	Allogeneic	Unknown	Unknown	LC	20	48 weeks	Phase 1–2	Randomized, parallel assignment, open label	NCT01342250	Completed		
11	2012	Bone marrow	Allogeneic	Hepatic artery	Unknown	LC (Alcohol)	40	96 weeks	Phase 2	Randomized, parallel assignment, open label	NCT01591200	Completed		
12	2012	Umbilical cord	Allogeneic	Peripheral vein	1.0 × 105/kg, 4 times	Liver failure (HBV)	120	48 weeks	Phase 1–2	Randomized, parallel assignment, open label	NCT01724398	Unknown		
13	2016	Bone marrow	Autologous	Portal vein	2.0 × 106/kg	LC	40	24 weeks	Phase 1–2	Non-randomized, parallel assignment, open label	NCT02943889	Not yet recruiting		
14	2009	Umbilical cord	Allogeneic	Portal vein or hepatic artery	Unknown	LC	200	48 weeks	Phase 1–2	Randomized, parallel assignment, single blind (subject)	NCT01233102	Suspended		
15	2009	Bone marrow	Autologous	Portal vein	Unknown	LC (HBV)	60	48 weeks	Phase 2	Non-randomized, parallel assignment, open label	NCT00993941	Unknown		
16	2010	Umbilical cord	Allogeneic	Hepatic artery	Unknown	LC	50	4 weeks	Phase 1–2	Randomized, parallel assignment, open label	NCT01224327	Unknown		
17	2013	Bone marrow	Autologous	Hepatic artery	1.0 × 106/kg	LC	30	12 weeks	Phase 3	Non-randomized, single group assignment, open label	NCT01854125	Enrolling by invitation		
18	2012	Umbilical cord	Allogeneic	Hepatic artery	1.0 × 106/kg	LC (HBV)	240	48 weeks	Phase 1–2	Randomized, parallel assignment, open label	NCT01728727	Unknown		
19	2013	Umbilical cord or bone marrow	Allogeneic	Peripheral vein	1.0 × 105/kg, 1.0 × 106/kg or 1.0 × 107/kg, 8 times	Liver failure (HBV)	210	72 weeks	Phase 1–2	Randomized, parallel assignment, open label	NCT01844063	Recruiting		
20	2016	Umbilical cord	Allogeneic	Peripheral vein	4 or 8 times	ACLF (HBV)	261	52 weeks	Phase 2	Randomized, parallel assignment, open label	NCT02812121	Not yet recruiting		
21	2010	Menstrual blood	Allogeneic	Peripheral vein	1.0 × 106/kg, 4 times	LC	50	48 weeks	Phase 1–2	Randomized, single group assignment, open label	NCT01483248	Enrolling by invitation		
22	2008	Bone marrow	Autologous	Hepatic artery	Unknown	LC	50	96 weeks	Phase 2	Randomized, parallel assignment, single blind (subject)	NCT00976287	Unknown		
23	2012	Bone marrow	Autologous	Hepatic artery	5 × 107/patient, 1 time or 2 times	LC (alcohol)	72	24 weeks	Phase 2	Randomized, parallel assignment, open label	NCT01875081	Completed	Histological improvement. Improvement in AST, ALT, ALP, γ-GTP, Child-Pugh score, and MELD score.	16
24	2014	Bone marrow	Autologous	Peripheral vein	Unknown	LC	10	24 weeks	Phase 1	Non-randomized, single group assignment, open label	NCT02327832	Recruiting		
25	2005	Bone marrow	Autologous	Hepatic artery	3.4 × 108/patient	Liver failure (HBV)	158	192 weeks	Phase 1–2	Case control, retrospective	NCT00956891	Completed	Improvement in Alb, T-Bil, PT, and MELD score.	
26	2009	Umbilical cord	Allogeneic	Peripheral vein	5.0 × 105/kg, 3 times	LC	45	48 weeks	Phase 1–2	Randomized, parallel assignment, open label	NCT01220492	Unknown	Improvement in Alb, T-Bil, and MELD score. Reduction of ascites.	17
27	2010	Bone marrow	Autologous	Portal vein	1.4–2.5 × 108/patient, 2 times	LC	2	48 weeks	Phase 1	Non-randomized, single group assignment, open label	NCT01454336	Completed	Transient improvement in MELD scores.	18
28	2007	Bone marrow	Autologous	Peripheral vein	(1.2–2.95 × 108) 1.95 × 108/patient	LC	27	48 weeks	Unknown	Randomized, parallel assignment, double blind (subject, outcomes assessor)	NCT00476060	Unknown	No beneficial effect.	19
29	2011	Bone marrow	Allogeneic	Hepatic artery and peripheral artery	1.0 × 106/kg (5.0 × 107 cells via the hepatic artery and the remaining cells via the peripheral vein)	Wilson’s disease	10	24 weeks	Unknown	Non-randomized, single group assignment, open label	NCT01378182	Completed		
30	2016	Umbilical cord or bone marrow	Allogeneic	Portal vein or hepatic artery	2.0 × 107/patient, 4 times	LC	20	48 weeks	Phase 1	Non-randomized, single group assignment, open label	NCT02652351	Recruiting		
31	2016	Bone marrow	Autologous	Hepatic artery	5 × 107/patient, 1 time or 2 times	LC (alcohol)	50	144 weeks	Phase 2	Randomized, parallel assignment, open label	NCT02806011	Enrolling by invitation		
32	2011	Umbilical cord	Allogeneic	Peripheral vein	1.0 × 106/kg, 3 times	Liver failure (AIH)	100	96 weeks	Phase 1–2	Randomized, parallel assignment, open label	NCT01661842	Unknown		
33	2009	Adipose tissue	Autologous	Unknown	Unknown	LC	6	24 weeks	Phase 1	Non-randomized, single group assignment, open label	NCT00913289	Terminated		
34	2012	Adipose tissue	Autologous	Hepatic artery	Unknown	LC	4	4 weeks	Unknown	Non-randomized, single group assignment, open label	NCT01062750	Completed		
35	2016	Umbilical cord	Allogeneic	Lobe	5.0 × 108/patient	LC	40	96 weeks	Phase 1–2	Randomized, parallel assignment, double blind (subject, outcomes assessor)	NCT02786017	Recruiting		
36	2011	Bone marrow	Unknown	Peripheral vein	5.0–50 × 106/kg	LC (PBC)	20	96 weeks	Phase 1	Randomized, parallel assignment, open label	NCT01440309	Unknown		
37	2011	Umbilical cord	Allogeneic	Peripheral vein	5.0 × 105/kg, 3 times	LC (PBC)	7	48 weeks	Phase 1–2	Randomized, parallel assignment, open label	NCT01662973	Unknown	Improvement in Alb, T-Bil, and MELD score. Reduction of ascites.	20
38	2010	Bone marrow	Allogeneic	Portal vein or hepatic artery	Unknown	Liver failure (HBV)	60	48 weeks	Phase 2	Non-randomized, parallel assignment, open label	NCT01221454	Unknown		
39	2010	Bone marrow	Allogeneic	Portal vein or hepatic artery	Unknown	LC	60	48 weeks	Phase 2	Non-randomized, parallel assignment, open label	NCT01223664	Unknown		
40	2010	Bone marrow	Autologous	Hepatic artery	(0.25–1.25 × 106) 0.75 × 106/patient	LC (HBV)	39	24 weeks	Phase 2–3	Non-randomized, parallel assignment, open label	NCT01560845	Unknown	Decrease in Th-17 cells, RORγt, IL-17, TNF-α, and IL-6. Increase in Tregs and Foxp3.	21

*LC* liver cirrhosis, *ACLF* acute-on-chronic liver failure, *HBV* hepatitis B virus, *HCV* hepatitis C virus, *AIH* autoimmune hepatitis, *PBC* primary biliary cholangitis, *MELD* Model for End-Stage Liver Disease, *AST* aspartate transaminase, *ALT* alanine transaminase, *ALP* alkaline phosphatase, *γ-GTP* gamma-glutamyl transpeptidase, *Alb* albumin, *T-bill* total bilirubin, *PT* prothrombin time, *PC* protein C, *ROR* RAR-related orphan receptor, *Foxp3* forkhead box *P3*, IL interleukin, *Th* T helper, *SMA* smooth muscle actin, *TGF* transforming growth factor, *TNF* tumor necrosis factor

**Fig. 2:**
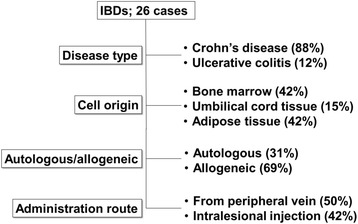
Summary of clinical trials in inflammatory bowel diseases

In IBDs, 26 trials are registered (Table 2), 23 of which are investigating the use of MSCs in Crohn’s disease (CD), and 3 of which are investigating the use of MSCs in ulcerative colitis (UC) [24–33]. More than 60% of trials are employing allogeneic MSCs, and in CD, more than 40% of the trials are evaluating intralesional injection into the fistula, which is the major and refractory complication of CD (Fig. 2).

**Table 2:** Clinical trials in inflammatory bowel diseases

No.	Start year	Cell source	Autologous/allogeneic	Administration route	Number of cells infused	Diseases	Number of patients	Follow-up period	Phase	Study design	ClinicalTrials.gov identifier	Status	Result	References
1	2006	Bone marrow	Allogeneic	Peripheral vein	8 × 106 cells/kg, 2 times or 2 × 106 cells/kg, 2 times	Crohn’s disease	10	4 weeks	Phase 2	Randomized, parallel assignment, open label	NCT00294112	Completed		
2	2007	Bone marrow	Allogeneic	Peripheral vein	Total of 6 × 108 cells/patient, 4 times or total of 12 × 108 cells/patient, 4 times	Crohn’s disease	98	24 weeks	Phase 3	Randomized, parallel assignment, double blind	NCT00543374	Completed		
3	2010	Adipose tissue	Autologous	Unknown	Unknown	Fistulizing Crohn’s disease	15	3 years	Phase 1–2	Non-randomized, single group assignment, open label	NCT01157650	Completed		
4	2015	Umbilical cord	Allogeneic	Peripheral vein	Unknown	Crohn’s disease	32	1 year	Phase 1–2	Randomized, parallel assignment, open label	NCT02445547	Completed		
5	2012	Bone marrow	Allogeneic	Peripheral vein	2 × 108 cells/patient, more than 4 times	Crohn’s disease	11	4 weeks	Phase 1–2	Non-randomized, single group assignment, open label	NCT01510431	Completed		
6	2010	Bone marrow	Allogeneic	Peripheral vein	2 × 106 cells/kg, 4 times	Crohn’s disease	15	6 weeks	Phase 2	Non-randomized, single group assignment, open label	NCT01090817	Completed	Improvement in CDAI, AQoL score. Decrease in CRP. Endoscopic improvement	24
7	2012	Bone marrow	Autologous	Peripheral vein	2 × 106 cells/kg, 5 × 106 cells/kg, or 1 × 107 cells/kg	Crohn’s disease	16	1 year	Phase 1	Non-randomized, single group assignment, open label	NCT01659762	Completed		
8	2010	Bone marrow	Allogeneic	Intralesional	1 × 107 cells/patient, 3 × 107 cells/patient, or 9 × 107 cells/patient	Fistulizing Crohn’s disease	21	12 weeks	Phase 1–2	Randomized, parallel assignment, double blind	NCT01144962	Completed	Local treatment with MSCs showed promotion of fistula healing. Lower MSC dose seemed superior.	25
9	2009	Adipose tissue	Autologous	Intralesional	3 × 107 cells/patient (in the event of incomplete closure at 8 weeks, a second injection was given that contained 1.5 times more cells than the f irst)	Fistulizing Crohn’s disease	43	8 weeks	Phase 1	Non-randomized, single group assignment, open label	NCT00992485	Completed	Local treatment with MSCs showed promotion of fistula healing.	26
10	2010	Adipose tissue	Allogeneic	Intralesional	2 × 107 cells/patient (in the event of incomplete closure at 12 weeks, an additional 4 × 107 cells were administered)	Fistulizing Crohn’s disease	24	24 weeks	Phase 1–2	Non-randomized, single group assignment, open label	NCT01372969	Completed	Local treatment with MSCs showed promotion of fistula healing.	27
11	2009	Adipose tissue	Autologous	Intralesional	1 × 107 cells/patient, 2 × 107 cells/patient, or 4 × 107 cells/patient	Fistulizing Crohn’s disease	10	4 weeks	Phase 1	Non-randomized, single group assignment, open label	NCT00992485	Completed	Local treatment with MSCs showed promotion of fistula healing. All patients with complete healing showed a sustained effect.	28
12	2009	Adipose tissue	Allogeneic	Intralesional	2 × 107 cells/patient (in the event of incomplete closure at 12 weeks, an additional 4 × 107 cells were administered)	Fistulizing Crohn’s disease	10	12 weeks	Phase 1–2	Non-randomized, single group assignment, open label	NCT00999115	Completed	Local treatment with MSCs showed promotion of fistula healing; 60% of patients achieved complete healing.	29
13	2009	Adipose tissue	Autologous	Intralesional	1 × 107 cells/cm^2^	Fistulizing Crohn’s disease	43	8 weeks	Phase 2	Non-randomized, single group assignment, open label	NCT01011244	Completed	In most cases, complete closure after initial treatment was well-sustained over a 24-month period.	30
14	2007	Bone marrow	Allogeneic	Peripheral vein	Total of 6 × 108 cells/patient, 4 times or total of 1.2 × 109 cells/patient, 4 times	Crohn’s disease	330	4 weeks	Phase 3	Randomized, parallel assignment, double blind	NCT00482092	Active		
15	2012	Adipose tissue	Allogeneic	Intralesional	1.2 × 108 cells/patient	Fistulizing Crohn’s disease	212	24 weeks	Phase 3	Randomized, parallel assignment, double blind	NCT01541579	Active	Local treatment with MSCs showed promotion of fistula healing.	31
16	2010	Bone marrow	Allogeneic	Peripheral vein	2 × 10^8 cells/patient, 3 times	Crohn’s disease	120	180 days	Phase 3	Non-randomized, single group assignment, open label	NCT01233960	Active		
17	2015	Adipose tissue	Autologous	Intralesional	Unknown	Fistulizing Crohn’s disease	10	62 weeks	Phase 2	Non-randomized, single group assignment, open label	NCT02403232	Recruiting		
18	2013	Bone marrow	Autologous	Intralesional	Unknown	Fistulizing Crohn’s disease	10	16 weeks	Phase 1	Randomized, parallel assignment, single blind	NCT01874015	Recruiting		
19	2015	Adipose tissue	Allogeneic	Peripheral vein	5 × 107 cells/patient, 7.5 × 107 cells/patient, or 1 × 108 cells/patient	Crohn’s disease	9	4 weeks	Phase 1	Non-randomized, single group assignment, open label	NCT02580617	Recruiting		
20	2013	Umbilical cord	Allogeneic	Peripheral vein	5 × 107 cells/patient or 1 × 108 cells/patient	Crohn’s disease	24	12 weeks	Phase 1–2	Non-randomized, single group assignment, open label	NCT02000362	Recruiting		
21	2013	Adipose tissue	Autologous	Intralesional	2 × 107 cells/patient	Fistulizing Crohn’s disease	20	2–24 months	Phase 1	Non-randomized, single group assignment, open label	NCT01915927	Recruiting		
22	Unknown	Bone marrow	Autologous	Peripheral vein	1–2 × 106 cells/kg	Crohn’s disease	10	6 weeks	Phase 1	Unknown	–	–	Three patients showed clinical response (decrease in CDAI).Three patients required surgery due to disease worsening.	32
23	2016	Bone marrow	Allogeneic	Intralesional	2 × 107 cells/patient	Fistulizing Crohn’s disease	20	7, 10, 16 months	Phase 1	Non-randomized, single group assignment, open label	NCT02677350	Not yet recruiting		
24	2015	Umbilical cord	Allogeneic	Peripheral vein	1 × 106 cells/kg, 3 times	Ulcerative colitis	30	24 weeks	Phase 1–2	Randomized, parallel assignment, single blind	NCT02442037	Recruiting		
25	2015	Adipose tissue	Allogeneic	Through a colonoscope	6 × 107 cells/patient	Ulcerative colitis	8	12 weeks	Phase 1–2	Non-randomized, single group assignment, open label	NCT01914887	Unknown		
26	2015	Umbilical cord	Allogeneic	First: peripheral vein, second: superior mesenteric artery	First: 3.8 ± 1.6 × 107 cells/patient, second: 1.5 × 107 cells/patient	Ulcerative colitis	80	12 weeks	Phase 1–2	Non-randomized, single group assignment, open label	NCT01221428	Unknown	Decrease in the median Mayo score and histology score. Improvement in IBDQ scores.	33

*CD* Crohn’s disease, *CDAI* Crohn’s Disease Activity Index, *AQoL* The Assessment of Quality of Life, *CRP* C-reactive protein, *IBDQ* Inflammatory Bowel Disease Questionnaire

## Clinical trials in liver diseases

### Background of liver diseases

Although the liver has high regenerative capacity, acute liver damage caused by viruses, drugs, alcohol, and autoimmune diseases, or chronic liver damage caused by hepatitis B or C virus, alcohol, non-alcoholic steatohepatitis (NASH), autoimmune hepatitis, and primary biliary cholangitis often cause liver failure [34]. The liver has a variety of functions, including metabolism of protein, sugar, and fat; detoxification; production of coagulation factors; and production of bile. Thus, during liver failure, several symptoms, including jaundice, edema, ascites, hepatic encephalopathy, and increased bleeding, can appear at the same time, resulting in life-threatening disease. In addition, during liver failure caused by chronic liver disease, accumulated liver fibrosis (i.e., liver cirrhosis) can cause portal hypertension, which often induces the varices, and long-term liver damage can cause gene abnormalities, leading to liver cancers. The ultimate therapy for liver failure is liver transplantation; however, only a small portion of patients with liver failure can receive liver transplantation due to the shortage of donor organs, invasiveness of operations, and economic reasons [35]. Revolutionary treatments, such as interferon-free treatment for hepatitis C and providing information regarding the importance of the daily lifestyle to prevent alcoholic liver disease and NASH, can potentially decrease the liver diseases; however, unmet needs to treat advanced liver failure will continue.

Advanced acute liver failure and chronic liver failure (liver cirrhosis) can be good targets for cell therapy. Since 2003, Terai et al. initiated autologous bone marrow cell infusion (ABMi) therapy against decompensated liver cirrhosis and confirmed the improvement of liver fibrosis and liver function [36–38]. However, due to the invasiveness of liver transplantation in patients with liver failure, minimally invasive procedures using specific cells, such as MSCs and macrophages [39–41], are now being developed, with a focus on MSCs. In the next section, we will describe recent reported results using MSCs registered at ClinicalTrials.gov.

### Effects of MSC therapy in liver disease from published papers

Animal experiments have shown that MSCs can have anti-apoptotic [42] and antioxidant effects in hepatocytes [43], and antifibrotic [44, 45], angiogenic [46], and immunosuppressive effects in T cells, macrophages, and dendritic cells [8]. In human clinical trials, all reports have shown that MSC injection is safe. Although the effects of cell therapy are not uniform, the majority of therapies have some beneficial effects; in contrast, in a few reports, treatment effects were not observed. For example, Kantarcioglu et al. [13] and Mohamadnejad et al. [19] injected bone marrow-derived MSCs into patients with liver cirrhosis and did not observe treatment effects. However, Kharaziha et al. [14] reported phase I–II clinical trials using autologous bone marrow-derived MSCs against liver cirrhosis with a variety of etiologies, and improvement of liver function was confirmed. Jang et al. and Suk et al. [12, 16] reported a pilot study and a phase II study using autologous bone marrow-derived MSCs injected through the hepatic artery against alcoholic liver cirrhosis, and improvement of histological liver fibrosis and liver function was confirmed. Xu et al. [21] reported trials using autologous bone marrow-derived MSCs against hepatitis B virus-associated cirrhosis and confirmed the improvement of liver function, the decrease of Th17 cells, and the increase of regulatory T cells. Xhang et al. [17] and Wang et al. [20] reported trials using allogeneic umbilical cord-derived MSCs in patients with chronic hepatitis B having decompensated liver cirrhosis and primary biliary cirrhosis, respectively. They confirmed improvement of liver function, particularly reduced ascites and recovery of biliary enzymes, respectively. Shi et al. [15] reported a trial investigating acute or chronic liver failure associated with hepatitis B virus and confirmed that MSCs significantly increased survival rates. From these reports, MSCs appeared to improve liver function; however, additional trials are needed to confirm these effects and to elucidate the mechanisms in more detail.

## Clinical trials in IBDs

### Background of IBDs

IBDs are chronic inflammatory disorders, including UC and CD. The pathogenesis of IBD is thought to be highly complex due to several factors, such as environmental factors, genetic predisposition, and inflammatory abnormalities [47]. UC is characterized by inflammation of the mucosal membrane of the colon continued from the rectum. Type 2 T helper cell (Th2) cytokine profile is associated with the pathogenesis of UC. In contrast, CD is a segmental, transluminal disorder that can arise within the entire gastrointestinal tract from the mouth to the anus. Th1 cells are associated with the pathogenesis of CD [48]. Furthermore, a recent report showed that Th17 cells are present in both UC and CD. Thus, mucosal CD4+ T cells are key mediators of the driving response [49]. Macrophages that produce tumor necrosis factor (TNF)-α have also been reported to be relevant in IBD. Imbalances in other cytokines, such as interleukin (IL)-1β, IL-6, IL-8, IL-10, IL-12, IL-17, IL-23, and transforming growth factor-β (TGF-β), are also detected during diseases [48]. Recent advancements in the development of drugs for IBD include drugs targeting TNF and new candidate drugs, such as antibodies against IL-6 [50] and IL-12/23 [51–53], small molecules including Janus kinase inhibitors [54], antisense oligonucleotides against SMAD7 mRNA [55], and inhibitors of leukocyte trafficking to intestinal sites of inflammation [56, 57]. However, some patients will fail to respond to current medical options, immunosuppressive agents, and anti-TNF biologicals. MSCs may be an effective option in these patients [9, 49]. In the next section, we will describe recently reported results using MSCs registered in ClinicalTrials.gov.

### Effects of MSC therapy in IBD from published papers

Eight CD trials and one UC trial have been published in ClinicalTrials.gov. Six papers describing CD are on trials treating fistula, and two papers are trials for luminal CD. Molendijk et al. [25] reported improved healing of refractory perianal fistulas using allogeneic bone marrow-derived MSCs. They administered these allogeneic MSCs locally and concluded that injection of 3 × 10^7^ MSCs appeared to promote the healing of perianal fistula. Panes et al. [31] reported a phase III randomized, double-blind, parallel-group, placebo-controlled study of complex perianal fistula using expanded allogeneic adipose-derived MSCs and confirmed the safety of the MSCs and the healing effects of MSCs on the fistula. Duijvestein et al. [32] reported a phase I study of refractory luminal CD using autologous bone marrow-derived MSCs and confirmed the safety and feasibility of MSC therapy. Forbes et al. [24] reported a phase II study using allogeneic bone marrow-derived MSCs for luminal CD refractory to biologic therapy. They administered 2 × 10^6^ cells/kg weekly for 4 weeks and found that allogeneic MSCs reduced the CD activity index (CDAI) and CD endoscopic index of severity (CDEIS) scores in patients with luminal CD refractory to biologic therapy. Hu et al. [33] reported a phase I/II study for severe UC using umbilical cord-derived allogeneic MSCs by combination injection through the peripheral blood and superior mesenteric artery with a 7-day interval. They confirmed the safety of MSCs and alleviation of diffuse and deep ulcer formation and severe inflammatory mucosa by MSCs.

## Safety of the MSC therapy

MSC therapy is associated with some concerns, such as adverse events related to infusion, tumor formation during the treatment of liver cirrhosis, and long-term observations of tumor formation. Regarding adverse events related to the infusion, Lalu et al. performed a meta-analysis of the safety of MSCs in clinical trials and showed that autologous and allogeneic MSC therapies were related to transient fever but not infusion toxicity, organ system complications, infection, death, and malignancies (Table 2) [5]. Regarding tumor formation during the treatment of liver cirrhosis, Peng et al. reported that no severe adverse events or no significant differences in tumor formation were detected compared with those in the control group during autologous bone marrow-derived MSC therapy for liver cirrhosis [58]. Regarding long-term observations of tumor formation derived from MSCs, Bahr et al. reported recent autopsy data from patients in a clinical trial of graft-versus-host disease (GvHD) who received MSC therapy between 2002 and 2007 and revealed no ectopic tissues, neoplasms, or donor-derived DNA [6].

## Conclusions

Many clinical trials of autologous and allogeneic MSCs have aimed to elucidate the effects and mechanisms of MSCs. MSCs can expand easily and can be obtained from medical waste, suggesting their applications in regenerative medicine for the treatment of liver diseases and IBDs. Recently, limitations of MSCs have been reported. For example, therapeutic effects were not long term and were affected by inflammatory condition [59, 60]. Thus, the results of ongoing clinical studies will be expected to provide further insights.

## Abbreviations

ABM*i*: Autologous bone marrow cell infusion
CD: Crohn’s disease
CD CDEIS: Endoscopic index of severity
CDAI: CD activity index
ECM: Extracellular matrix
GvHD: Graft-versus-host disease
IBD: Inflammatory bowel disease
IL: Interleukin
MSCs: Mesenchymal stem cells
NASH: Non-alcoholic steatohepatitis
TGF-β: Transforming growth factor
TNF: Tumor necrosis factor
UC: Ulcerative colitis
